# Controlled temperature-induced dormancy leads to predictable microbial recovery in the temperate coral microbiome

**DOI:** 10.3389/fmicb.2026.1799468

**Published:** 2026-05-07

**Authors:** Anya L. Brown, Meriel J. McGovern, Alicia Schickle, Koty H Sharp, Amy Apprill

**Affiliations:** 1 Bodega Marine Lab, University of California, Davis, Bodega Bay, CA, United States; 2 Woods Hole Oceanographic Institution, Woods Hole, MA, United States; 3 Center for Economic & Environmental Development/Department of Biology, Marine Biology, and Environmental Science, Roger Williams University, Bristol, RI, United States; 4 Molecular Biology, Princeton University, Princeton, NJ, United States

**Keywords:** *Astrangia poculata*, community trajectory, coral, microbiome, quiescence

## Abstract

**Introduction:**

Dormancy is a common response to harsh conditions across the tree of life. Increasingly, the animal microbiome is suggested to mediate dormancy, including onset, maintenance and exit or recovery periods. However, it is unclear what triggers dormancy and the consistency of microbial patterns across populations. Because it regularly undergoes dormancy during winter in the northernmost part of its range, the temperate coral *Astrangia poculata* can serve as a model for studying coral-microbiome dynamics during environmental stress and recovery.

**Methods:**

Here, we experimentally manipulated temperature to induce dormancy and measured the microbial community shifts associated with dormancy and during the days that the corals were exiting dormancy in two populations.

**Results:**

Our treatments successfully induced dormancy, which was maintained through low temperatures. We found consistent and predictable shifts in the microbiome during dormancy and during the recovery period while corals exited dormancy.

**Discussion:**

We suggest *Sulfitobacter*, a key genus in tropical corals, may be involved in early recovery in the assembly of the microbiome. Broadly, our results highlight that the inducible, consistent, and persistent microbial restructuring associated with *Astrangia* dormancy can be applied more generally to tropical coral recovery from stress.

## 1 Introduction

Species across the tree of life (e.g., Eubacteria, Animalia) have evolved to undergo dormancy to survive periods of harsh environmental stress. Dormancy represents a resting state, in which metabolic functions are depressed (Hand, 1991; Cáceres, 1997). The interplay between dormancy and the microbiome has recently been explored in a wide range of animals, revealing an intimate association that may be integral to the long-term health of host-microbial meta-organisms. A deeper understanding of the patterns and cycles of dormancy-driven shifts in the microbiomes, or the microbiomes’ role in mediating the animal dormancy period, is likely a critical component of organismal and ecosystem-level responses to climate change.

In many species, host-associated microbiomes shift throughout the course of the animal host’s dormancy period, including in squirrels (Carey et al., 2013; Regan et al., 2022), brown bears (Sommer et al., 2016), parasitoid wasps (Dittmer and Brucker, 2021), crickets (Ferguson et al., 2018) and aquatic crustaceans (Mushegian et al., 2018). Indeed, the ground squirrel gut microbiomes are restructured during dormancy, modulated by variation in diet (active vs. not active feeding) throughout the year (Carey et al., 2013). These alterations can be long-lasting; for example, parasitoid wasps show microbial community alterations during and following dormancy (i.e., diapause) in larval stages that persist into adulthood (Dittmer and Brucker, 2021). Exiting dormancy is analogous to recovery, and these microbial community shifts may enhance a host species ability to tolerate varying environmental conditions (Reshef et al., 2006).

In aquatic invertebrates, the onset of dormancy or quiescence is often associated with harsh environmental conditions, such as winter temperatures in temperate climates. The only coral known to regularly undergo dormancy in response to winter stress is the temperate scleractinian coral *Astrangia poculata*, which annually enters into a winter dormancy referred to as “quiescence” in the northernmost portion of their geographic range (Grace, 2017; Brown et al., 2022). Quiescent *A. poculata* show a different morphological and behavioral phenotype compared to non-dormant corals: they retract their tentacles, form a puffed-up ring around their oral disc, do not respond to tactile stimulation, exhibit reduced or ceased feeding, and their growth rate slows (Grace, 2017). Emergence from dormancy (i.e., recovery) includes re-activation of normal feeding and tentacular response and function.

A recent field survey suggests the *A. poculata* microbiome varies in composition before, at the onset, during, and after winter-induced dormancy (Brown et al., 2022). This study showed that corals remained dormant for over 2 months and experienced microbial community composition shifts that persisted as the corals emerged from dormancy. Microbes associated with nitrogen cycling increased in relative community abundance during dormancy, suggesting a replacement of host nutrition while the corals were dormant (Brown et al., 2022). Additionally, microbial alpha diversity declined during dormancy, compared to before and after dormancy, associated with a shedding of copiotrophic bacteria before dormancy and a re-colonization by bacteria following dormancy. During the recovery period following dormancy, the microbiome shifted to a new state (different from the pre-dormancy community) and similar to those previously documented in spring-time microbiomes of *A. poculata*, representing shifts that may be adaptive (Reshef et al., 2006; Sharp et al., 2017) for the coral.

*Astrangia poculata*’s feeding and physiological responses are limited during quiescence (Grace, 2017), and *A. poculata* genomic sequencing has revealed expansion of gene families involved in feeding suppression and sleep promotion that likely allow for this dormancy response and the species’ subsequent range expansion to habitats with extreme seasonal cold thermal stress (Stankiewicz et al., 2025). The associated microbial community-wide changes during quiescence likely represent a response to alterations in the holobiont (e.g., host and microbes, such as through declined host metabolic activity and subsequent production of regulatory host metabolites), to the surrounding environment (e.g., temperature, shifts in seawater composition), or a combination of both. Although the exact triggers of onset and emergence from dormancy are not known, field surveys suggest that *A. poculata* in the field start to go dormant after temperatures reach 5 °C and are sustained at or below that temperature for multiple days (Grace, 2017; Brown et al., 2022), although emergence temperature differs slightly between Massachusetts (at 5 °C) and Rhode Island (just above 5 °C, Grace, 2017; Brown et al., 2022). Further, though temperature is likely an important trigger for dormancy in *A. poculata*, it remains unclear whether dormancy-associated microbiome shifts are driven by changes in the *A. poculata* holobiont, by temperature, or by other factors in the environment. Because corals exhibit physiological changes according to their geographic-specific temperature exposure (McClanahan et al., 2020), it is not known if host activation and exiting of dormancy, and microbiome shifts will occur simultaneously in *A. poculata* individuals across different locations. To determine whether temperature-induced dormancy in the laboratory leads to microbial reshuffling in patterns similar to those previously observed in the field, and to identify the influence of geographic origin of the corals on dormancy-associated microbiome shifts, we conducted a lab-based manipulative experiment using corals from two geographically separated populations (Rhode Island and Woods Hole, Massachusetts, USA). We expected (a) microbial community composition to change due to dormancy, and that the new community structure would persist after dormancy; (b) an increase in microbes associated with nutrient cycling during dormancy, and an increase in microbes in the breakdown of organic materials as corals begin to emerge, or recover, from dormancy, and (c) differences in host and microbial community response to dormancy according to geographic collection location.

## 2 Methods

### 2.1 Experimental set up

**Figure 1:**
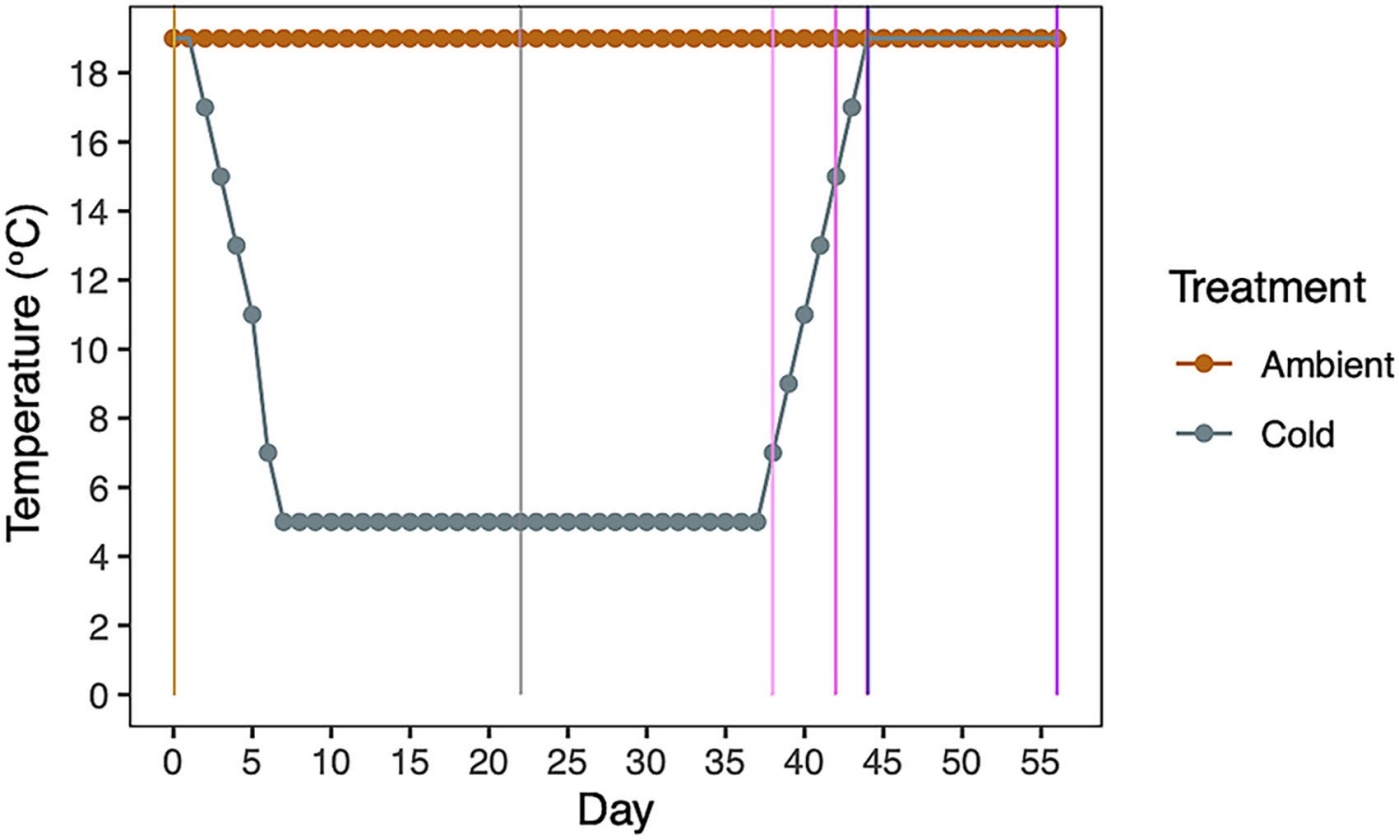
Temperatures in the ambient and cold ramping treatments. Following Day 1, corals in the cold treatment were lowered 2 °C per day until 5 °C was reached (on Day 7). Temperature was held at 5 °C until Day 37. Ambient corals were kept at 19 °C for the duration of the experiment. Colored lines intersecting the temperatures indicate time points when samples were taken before (orange), during (gray), and as temperatures ramped up (purple hues).

Aposymbiotic *A. poculata* colonies were collected via SCUBA from Fort Wetherill State Park, Jamestown Rhode Island (RI) from 10–15 m, and from under the dock at the Woods Hole Oceanographic Institution, Woods Hole, Massachusetts (MA) (18 m) in March 2021. The colonies were acclimated in the Roger Williams University laboratory aquatic systems at 19 °C until experiments began (May 2021). At the start of the experiment, forty *A. poculata* colonies were divided evenly into two different recirculating raceway systems (*n* = 10 per geographic origin, per raceway). Each colony was then placed in an individual, labeled, tri-pour beaker with direct seawater inflow from the system to each individual beaker to minimize cross-contamination of seawater among replicate colonies. The ambient (control) treatment was held at a constant 19 °C. To induce and maintain dormancy, we employed a temperature ramping treatment (cold treatment). The cold treatment started at 19 °C and temperature was lowered 2 °C a day until reaching 5 °C (over 1 week, reached 5 °C by Day 7), was held at 5 °C (for 4 weeks, until Day 37), and then temperatures were increased 2 °C per day to 19 °C (over 1 week, until Day 43, Figure 1). Corals were then held at 19 °C for an additional two weeks (until Day 57). In each system, seawater temperature was maintained and monitored via an in-line water chiller (Aqua-Logic, Inc.) and digital temperature controller (Aqua-Logic, Inc.).

### 2.2 Visual assessment of *A. poculata* colonies

To determine the host-level response to the treatment (ambient versus cold-inducing dormancy), and to help determine when corals went into dormancy, the percent of each colony retracted and polyp responsiveness were measured. The percent of the colony retracted was visually determined as an estimate of the whole colony with polyps pulled into the skeleton. Percent colony retraction over time between populations and treatment (cold versus ambient temperatures) was analyzed using a linear mixed effects model with fixed effects of populations and treatment and time, and in which coral colony was treated as a random intercept using the lme4 (Bates et al., 2015) and lmerTest (Kuznetsova et al., 2015) packages in R.

Time to dormancy using colony retraction and polyp responsiveness was compared between populations and treatments. Polyp responsiveness was determined by tapping firmly on the exterior of each tri-pour beaker with a metal instrument. Colonies were scored as “responsive” if any polyp exhibited movement. Time to dormancy was scored as the first day of two successive days when corals were unreponsive or when all tentacles on a colony were retracted. Time to recovery was analyzed with each metric, starting on day 37 when temperatures rose towards ambient in the cold treatment. Coral “recovery” was determined by consecutive days of polyp response and the first of two days in a row when corals were retracted less than 100%. We analyzed the time to dormancy and time to recovery using time to event analysis in R with the survival and ggfortify packages (Therneau, 1997; Tang et al., 2016) comparing treatments, with separate analyses for each response (retraction and polyp touch response) and each population.

### 2.3 Sampling of *A. poculata* mucus and seawater

Coral mucus samples for microbiome analysis were collected every other day during ramp-down and ramp-up periods, weekly during the 4 weeks of quiescence, and weekly for two weeks post-quiescence. Mucus was collected from each colony by swabbing each coral colony surface colony surface with a sterile flocked swab (HydraFLOCK, Puritan Medical Products, Guilford, ME) 15 times across the coral’s diameter (encompassing the whole coral surface). After collection from a colony, the swab was placed in a sterile 2 mL cryovial tube and kept on ice until the sampling was complete. After sampling, the tubes were stored in a −80 °C freezer until DNA extraction.

Seawater samples for microbiome analysis were taken during each coral mucus sampling. The incoming seawater (240 mL) to the coral cup (representing microbes present in the seawater system, not the cup where corals were housed) was collected with a 60 mL sterile syringe. Water was pushed through the syringe onto a Sterivex™ cartridge (0.2 μm pore size). These cartridges were wrapped in aluminum foil and immediately stored in a −80 °C freezer until extraction (*n* = 4 per treatment, except for Day 42, when *n* = 2 per treatment).

### 2.4 DNA extraction

Coral mucus swabs (*n* = 8 per treatment/population) and seawater filters (2–4 per treatment) were analyzed across 6 time points that spanned the experiment, but concentrated on the period surrounding the temperature increase in the cold treatment (Days 1, 22, 38, 42, 44, 53). DNA extractions for coral mucus samples and Sterivex filters were performed using the protocol for the Qiagen PowerBiofilm DNA isolation kit (Qiagen, Inc., Germantown, MD). For the coral mucus swabs, 350 μL of solution MBL and 100 μL of solution FB were added to each cryovial containing a swab, and tubes were capped and gently agitated. Flame-sterilized forceps were used to transfer each swab, shaft down, into UV sterilized microcentrifuge tubes. The tubes were centrifuged at 13,000 g for 1 min. After the swabs were discarded and the liquid from the tubes and the cryovials was transferred to the bead tubes, the manufacturer’s protocol was followed. For the seawater samples, each sterivex cartridge was cut open using sterilized bone cutters. The filter was removed and cut up using a sterilized razor blade and tweezers and added directly to the bead tube with 350 μL of solution MBL and 100 μL of solution FB and vortexed briefly. The remaining steps of the manufacturer’s protocol were then followed. We also extracted blank samples with identical methods (sterile swabs, kit blanks, filter blanks, *n* = 5 per blank type).

### 2.5 Library preparation and sequencing

Samples were prepped for 16S rRNA gene sequencing using standard dual indexing (Kozich et al., 2013) on the V4 region with primers that target bacteria and archaea (515FY, 806RB; Apprill et al., 2015; Parada et al., 2016). The PCRs (25 μL) were performed in duplicate for each sample using 14.75 μL of PCR grade water, 5 μL of GoTaq Flexi 5x buffer (Promega Corporation), 2.5 μL of 25 mM MgCl_2,_ 0.5 μL of each 10 μM forward and reverse primers, 0.5 μL of 10 mM dNTPs, and 0.25 μL GoTaq DNA polymerase (Promega) and 1 μL of the template DNA. We also included a negative PCR control (1 μL of PCR grade water added instead of template) and a positive control mock community (obtained through BEI Resources, NIAID and NIH as part of the Human Microbiome Project: Genomic DNA from Microbial Mock Community B [even, low concentration], v5.1L, HM-782D). The thermocycler conditions for corals were: an initial denaturation step of 95 °C for 2 min, and then 35 cycles of 95 °C (20s), 55 °C (15 s), 72 °C (5 min), and a final elongation step of 72 °C for 10 min. The water samples were treated as above, except using 28 cycles. For sequencing, PCR negative controls were pooled from each PCR.

Each coral PCR reaction was visualized on a 1.5% agarose gel to assure quality and to excise the correct band, determined by the location of the positive control and DNA ladder (~400 bp). Gels were extracted and purified with the MinElute Gel Purification kit using the manufacturer’s protocol (Qiagen Inc., Germantown, MD). Water samples were visualized on a 1% agarose gel to verify amplification, and the PCR products were then purified and concentrated using the MinElute PCR Purification Kit (Qiagen) following the manufacturer’s protocols. Each purified product was quantified on qubit fluorometer, diluted to 1 ng μl^−1^ and then pooled at 5 ng of purified product per sample. The pooled libraries (4 total) were sequenced on an Illumina MiSeq for 250 bp paired end sequencing. Raw files are in the SRA archive, accession numbers under BioProject PRJNA1440488; accession numbers SRX32740007-SRX32740256.

### 2.6 Bioinformatics

Bioinformatics were completed on the demultiplexed forward and reverse reads with the dada2 pipeline (v 1.16.0; Callahan et al., 2016) for quality control, merging reads and assigning ASVs (Amplicon Sequence Variants). For each MiSeq run, the quality of the forward and reverse reads were visually assessed to determine the cut off values for the filter and trim step (the approximate number of base pairs in which the quality scores fall below 30). The filter and trim parameters were: filterAndTrim (fnFs, filtFs, fnRs, filtRs, truncLen = c(240, 150), maxN = 0, maxEE = c(2,2), rm.phix = TRUE, compress = TRUE, multithread = TRUE). Error rates were computed for each run and used to infer unique sequences. Reads were then merged and four ASV tables were created. ASV tables were then merged, chimeras were checked and removed. We assigned taxonomy using the SILVA v138.1 (Quast et al., 2013; Yilmaz et al., 2014; Callahan et al., 2016) database and visually verified the retrieval of expected taxa from mock communities.

The ASV table, taxa table and associated metadata were loaded into phyloseq (McMurdie and Holmes, 2013) for additional quality control and analysis. The decontam package (Davis et al., 2018) was used for removing taxa associated with the controls using the prevalence of taxa or presence versus absence of taxa in the controls (3 swab blanks, 1 filter blank, 3 extraction blanks, 4 PCR negative and 4 positive controls) versus in the true samples based on chi squared test (at a threshold of 0.01). ASVs associated with chloroplasts or mitochondria were removed as well. Samples associated with controls were removed from the dataset for downstream analyses.

### 2.7 Alpha diversity

Because all samples reached an asymptote when rarefactions curves were visualized, coral and water samples were not rarefied (Supplementary Figure S1). Alpha diversity was calculated in three ways (richness, exponentiated Shannon, Inverse Simpson) as Hill numbers (Alberdi and Gilbert, 2019). Each diversity measure was analyzed with separate mixed effects generalized linear models comparing factors population (RI versus MA), time point (Day 1, 22, 38, 42, 44, 53) and treatment (ambient versus cold) using the lm4 package (Bates et al., 2015). Coral identity was treated as a random effect. Significance was assessed using an analysis of deviance. Each population was tested separately to determine within population differences in treatment and time. Pairwise differences between time and treatment were determined by Tukey *Post hoc* tests using the agricolae package in R (De Mendiburu, 2021). Water samples were examined using the combined and separate effects of treatment and time point and analyzed using linear models for each metric separately.

### 2.8 Beta diversity

#### 2.8.1 Beta dispersion

Beta diversity was calculated using beta dispersion (within-treatment variation) and community composition (across-treatment variation) differences. The betadisper function in vegan, which measures the distance between samples and their group centroid, was used to understand within-treatment variability (Oksanen et al., 2016). For coral samples, Bray–Curtis dissimilarity indices were used to make dissimilarity matrices based on the relative abundances of reads within a time point, population and treatment. The indices were used to compute beta-dispersion within each time point, population and treatment. For water samples, the same analyses as above were performed using time point and treatment to compute the Bray–Curtis dissimilarity matrices and beta dispersion, and then using linear mixed effects models as above.

#### 2.8.2 Community composition

Using the Bray–Curtis dissimilarity distance matrices described above, coral and water community composition differences were compared using PERMANOVA from the vegan package (Oksanen et al., 2016). For corals, we tested for the single and interactive effects of treatment (cold vs. ambient), day (T0, T22, T38, T42, T44, T57) and population (Rhode Island versus Woods Hole), with a blocking effect for each coral colony sampled. We then examined the pairwise comparisons for the significant interaction (e.g., the three-way interaction) using the pairwise adonis package (Martinez Arbizu, 2017). To understand differences in the community composition of the seawater microbiome, we compared the fixed effects of treatment and time using a PERMANOVA.

### 2.9 Specific microbial shifts and potential functional shifts

At each time and for each population, corals were compared between the ambient and cold treatment using separate DeSeq2 analyses (Love et al., 2014). Geometric means were used to estimate sizefactors and the local fit for estimating dispersion and alpha was set to 0.001. Putative functions of taxa were assigned using the FAPROTAX database (Liang et al., 2020) to identify differences in functional potential between the treatments and time points. Additionally, to visually compare seawater samples and the coral microbial samples, the significantly enriched ASVs on corals were plotted for both sample types in each treatment. All results and plots were analyzed and created in R v 4.2.2 (R Core Team, 2022).

## 3 Results

### 3.1 Coral host

**Figure 2:**
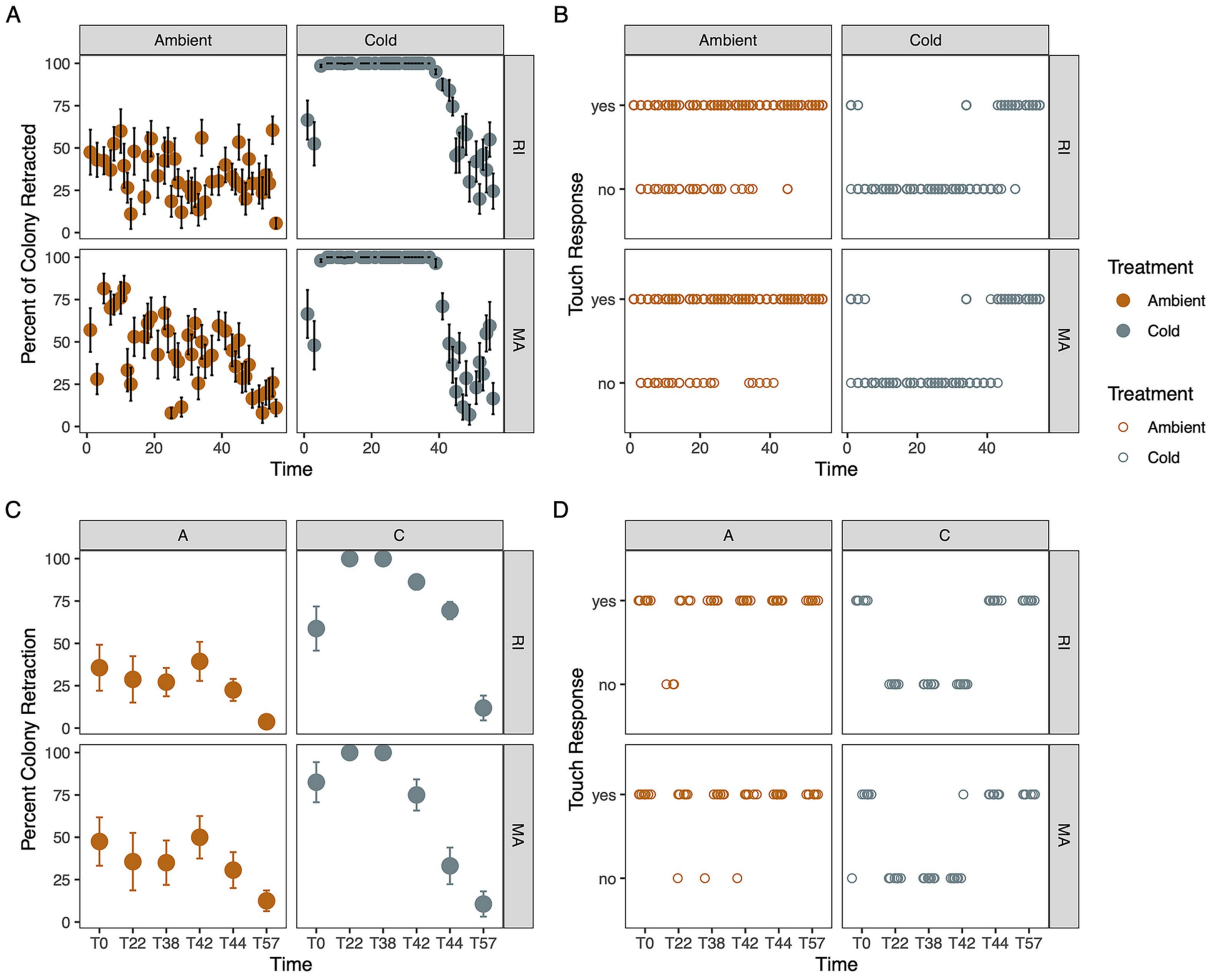
Host responses to treatments as indicators of coral dormancy. **(A)** Mean ± SE percent colony retraction of ambient and cold treatment corals **(B)** Polyp response to tapping. If all corals were retracted (100%), this indicates corals went into dormancy. **(C)** Percent colony retraction and **(D)** polyp response to tapping for corals that were sampled for their microbiome. When corals consistently did not respond to tapping, corals were considered dormant.

All cold treatment corals exhibited signs of dormancy by day 7 at 5 °C, and no corals in the ambient treatments entered dormancy (Figure 2, Supplementary Figure 2, survival analyses *p* < 0.001). Coral individuals in the cold treatment started to enter dormancy between days 3 and 5, when temperatures were decreased from 15 to 11 °C (Figure 2). Corals from Rhode Island and Massachusetts went into dormancy within the same number of days. As we increased the temperatures, corals in the cold treatments across both populations began to open their polyps and extend tentacles when temperatures reached 13 °C (Day 41; Figures 2A,C; Supplementary Figure 3; survival analysis, for each population). Massachusetts corals started to respond to touch when temperatures reached 17 °C on Day 43 (*p* < 0.05), and RI corals’ touch response started at 13 °C, Day 41 (Figures 2B,D; Supplementary Figure S3, *p* < 0.05). By 19 °C (Day 45), all corals were out of dormancy and actively responded to touch (Figures 2B,D, linear model, <0.001).

### 3.2 Coral microbiome

Sequences from 250 specimens, including 191 corals (*n* = 8 per population, treatment and time period; *n* = 7 from Rhode Island on Day 38 in ambient treatment), 44 water samples and 15 controls resulted in a total of 19,884,181 reads and 14,106 Amplicon Sequence Variants (ASVs). ASVs associated with the controls (63 ASVs) and chloroplasts and mitochondria (992 ASVs) were removed. The final dataset contained 16,903,709 reads, 12,943 ASVs and 235 samples. The dataset had a mean and standard deviation of 71930.68 ± 63524.71 reads per sample, and a range of 9,777–466,796 reads across coral and water samples.

### 3.3 Alpha diversity

**Figure 3:**
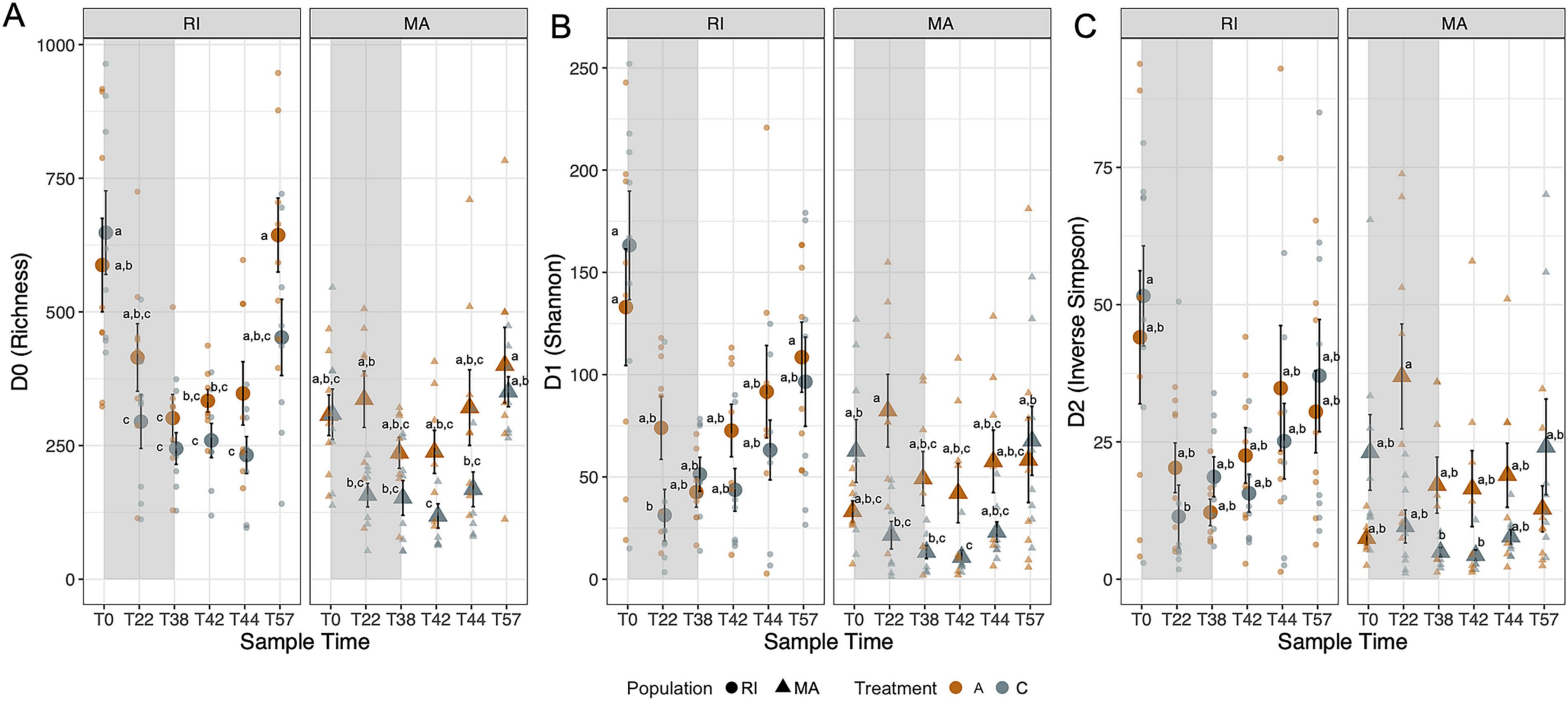
Alpha diversity measures throughout the experiment by coral field site origin, including Richness (D0) **(A)**, exponentiated Shannon diversity (D1) **(B)**, and Inverse Simpson (D3) **(C)**. Large points represent mean ± SE, and small points represent the raw values. Colors indicate treatment, gray represents cold treatments and orange ambient treatments. Shape represents population (circles are Rhode Island, RI, and triangles represent Massachusetts, MA). Letters indicates significant differences between time and treatment within a population. Points with different letters indicate significant differences (*p* < 0.05) based on Tukey HSD test. Shaded region represents time period while corals were in dormancy.

Compared to ambient corals, alpha diversity decreased in dormant corals (Day 22, Tukey HSD *p* < 0.001, Figure 3, Table 1, Tukey HSD in Supplementary Table S1), and increased when corals were out of dormancy, reaching diversity measurements similar to initial, pre-quiescence alpha diversity (day 57). Rhode Island corals contained higher microbial alpha diversity than Massachusetts (*p* < 0.05; Table 1, TSD results in Supplementary Table S1). Diversity, particularly evenness metrics like D1 and D2, (Figures 3B,C) decreased while corals were dormant and remained low even as corals exited dormancy (Time; *p* < 0.05, Supplementary Table S1).

**Table 1:** Coral microbial diversity comparisons between treatment, populations and time.

Response	Factor	χ ^2^	DF	*p*
Observed	Time	**103.82**	**5**	**<0.001**
	Treatment	**10.96**	**1**	**<0.001**
	Population	**26.00**	**1**	**<0.001**
	Time × Treatment	*10.01*	*5*	*0.07*
	Time × Population	**19.81**	**5**	**<0.001**
	Treatment × Population	0.06	1	0.81
	Time × Treatment × Population	3.39	5	0.64
Shannon	Time	**39.84**	**5**	**<0.001**
	Treatment	**17.46**	**1**	**<0.001**
	Population	**4.97**	**1**	**0.03**
	Time × Treatment	8.52	5	0.13
	Time × Population	**21.93**	**5**	**<0.001**
	Treatment × Population	0.51	1	0.47
	Time × Treatment × Population	6.35	5	0.27
Inverse simpson	Time	**20.19**	**5**	**<0.001**
	Treatment	**14.69**	**1**	**<0.001**
	Population	1.58	1	0.21
	Time × Treatment	**12.12**	**5**	**0.03**
	Time × Population	**22.72**	**5**	**<0.001**
	Treatment × Population	0.48	1	0.49
	Time × Treatment × Population	6.42	5	0.27
Beta dispersion	Time	0.13	1	0.72
	Treatment	**14.52**	**5**	**0.01**
	Population	**19.25**	**1**	**<0.001**
	Time × Treatment	9.11	5	0.10
	Time × Population	1.24	1	0.27
	Treatment × Population	**23.23**	**5**	**<0.001**
	Time × Treatment × Population	*10.82*	*5*	*0.06*

df, degrees of freedom; bold type indicates significant differences. Italics refer to marginally significant *p* values > 0.05 and less than 0.1.

### 3.4 Beta dispersion

**Figure 4:**
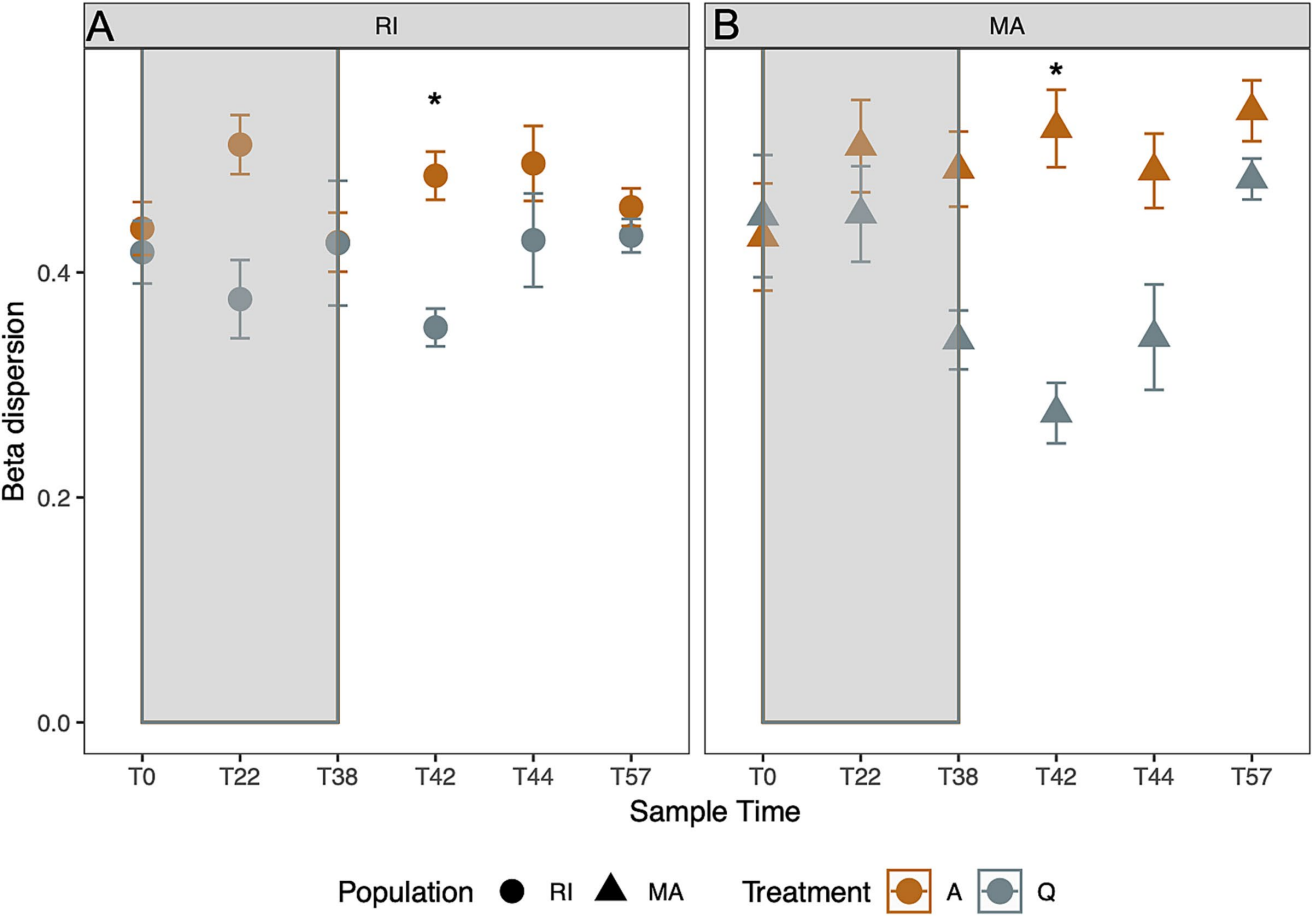
Variation in beta dispersion over time. Points represent mean ± SE in **(A)** Rhode Island (RI) and **(B)** Massachusetts (MA) coral microbiomes. As corals emerge from dormancy (Day 42), there is a significant decrease in dispersion (star indicates *p* < 0.05, Tukey HSD). Colors refer to treatment (orange indicates ambient, and gray indicates cold treatment).

Coral mucus microbiome beta dispersion decreased in the cold treatments while the corals were dormant, and increased after 57 days (Figures 4A,B; time × treatment: *p* < 0.001). For both MA and R populations, dispersion was lowest at day 42 in the cold treatment (Figures 4, *p* < 0.05, Tukey HSD). This low dispersion coincided with corals exiting the dormant state during the early recovery period.

### 3.5 Community composition

**Figure 5:**
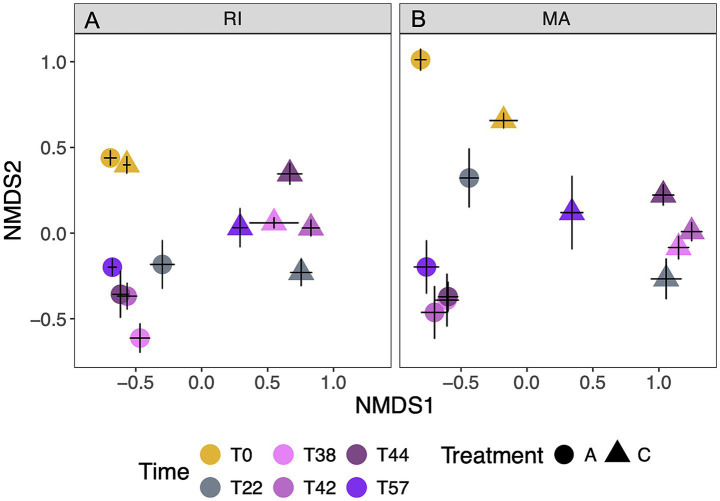
NMDS analysis demonstrating shifts in microbial community composition over time in **(A)** Rhode Island (RI) and **(B)** Massachusetts (MA). Points represent the nMDS plot mean ± SE location in NMDS space of corals (*n* = 8) within a treatment at each time point. Triangles represent corals exposed to the cold (C) treatment, and circles represent corals exposed to the ambient (A) treatment. Colors represent time point. Starting at T38, temperatures rose in the cold treatment until reaching ambient conditions (T44).

Cold treatments led to a significant shift in microbial composition associated with dormancy (Figure 5, *p* < 0.0001). Communities between Rhode Island and Massachusetts were initially different from each other (pairwise difference at T0, *p* < 0.05). For both coral populations, the cold treatment led to consistently different communities compared to the ambient treatment corals over time. The community composition differences between treatments persisted even as the cold-treatment corals exited dormancy. There were slight differences in how microbial communities progressed across time points between the populations and treatments. Massachusetts coral microbial communities shifted in composition between days 38–44, whereas the corals in ambient conditions remained consistent (corals; *p* < 0.05, see Supplementary Table S2 for full pairwise comparison results). However, corals from Rhode Island were similar between treatments on days 38 and 42 (*p* > 0.05), and differentiated from day 42–44 onwards (*p* < 0.05). Between populations, the microbial community composition of Rhode Island and Massachusetts corals were similar during quiescence (pairwise differences within a time point, day 22, *p* = 0.5), however as the temperatures increased (day 38, 42, 44,), the surface microbiome from each population diverged (*p* < 0.05), then converged by the last day of sampling (p > 0.05). For both populations, after dramatic initial changes, communities did not return to the same communities as they began, nor did communities converge to the same community composition as corals that remained in ambient conditions, even following 2 weeks held at the same temperature (*p* < 0.01, Table 2).

**Table 2:** PERMANOVA results for coral microbial communities compared between treatment, time and population.

Factor	DF	SS	*R* ^2^	*F*	*p*
Time	5	8.21	0.12	6.96	0.001
Treatment	1	9.22	0.13	39.06	0.001
Population	1	2.33	0.03	9.87	0.001
Time × Treatment	5	6.05	0.09	5.13	0.001
Time × Population	5	2.11	0.03	1.79	0.001
Treatment × Population	1	1.14	0.02	4.83	0.001
Time × Treatment × Population	5	1.71	0.02	1.45	0.001

DF, degrees of freedom; SS, sum of squares.

### 3.6 Significant microbial taxa and inferred functional microbiome changes

**Figure 6:**
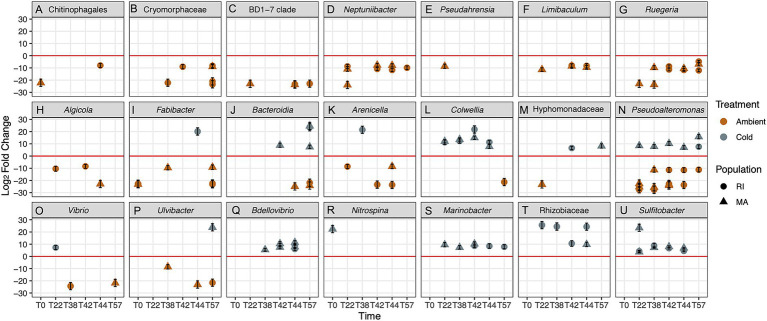
**(A–U)** Select taxa that were significantly different between ambient and cold treatments for RI and MA corals based on the DeSeq analysis. Values above zero indicate enrichment of taxa in the cold treatments, and below zero indicate enrichment in the ambient treatment, enrichment direction is also denoted by color. Shapes indicate population. Full taxa list of significantly different ASVs is in the supplement (Supplementary Table S3 in File S2). Multiple points indicate multiple ASVs within the genus.

During each time point, we found between 10 and 79 ASVs that differed significantly between cold and ambient treatments across both populations (Figure 6; see Supplementary Table S3 for all ASVs,). ASVs enriched in the ambient treatment corals include Chitonophagales, *Pseudarensia*, *Ruegaria*, *Algicola* (Figures 6A–H). ASVs enriched in cold treatment corals include *Bdellovibrio*, *Nitrospina*, *Marinobacter*, Rhizobiacea and *Sulfitobacter* (Figures 6Q–U). ASVs of the same genus that differed in their response in cold and ambient conditions include Bacteroidia *Arencella*, *Colwellia*, Hyphomonodaceae, *Pseudoalteromonas*, *Vibrio* and *Ulvibacter* (Figures 6I–P).

**Figure 7:**
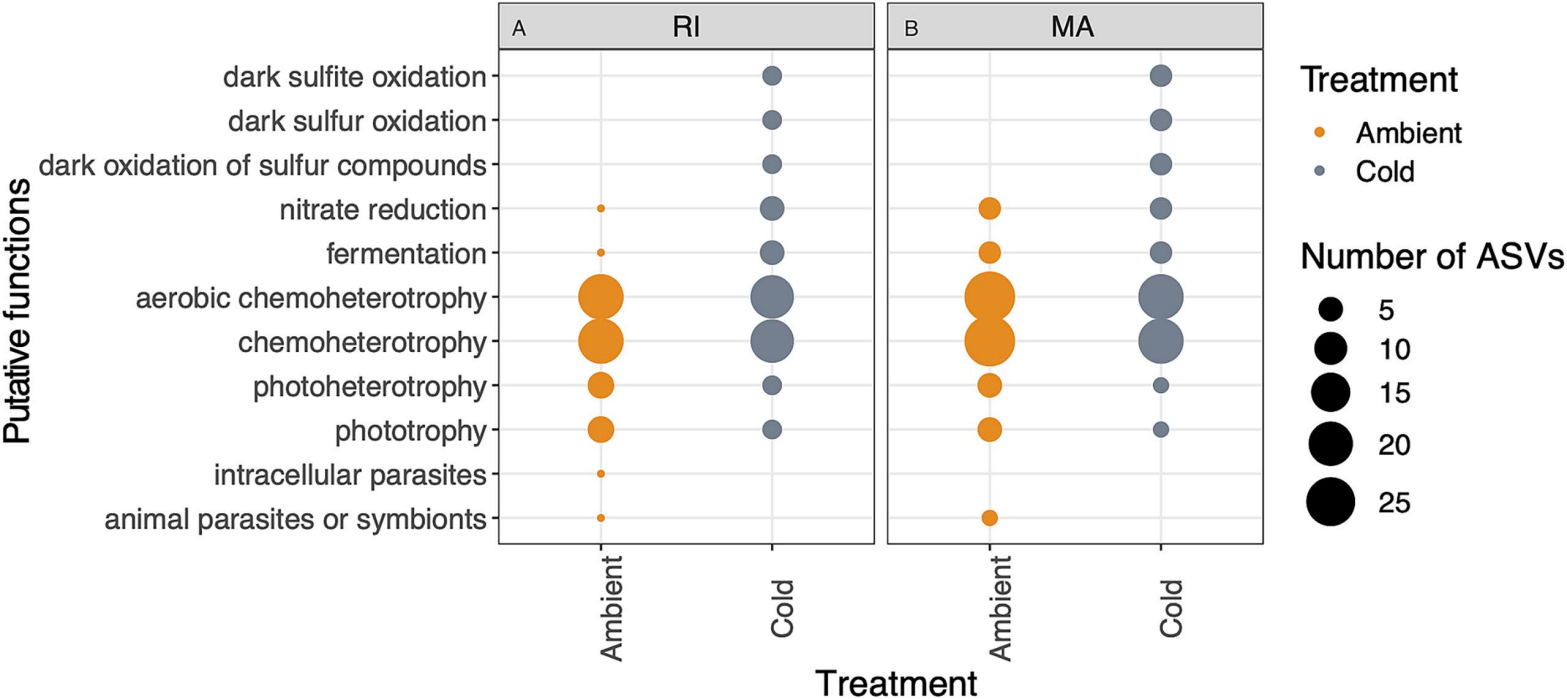
Putative microbial functions based on the FAPROTAX database of ASVs that were enriched in ambient or cold treatments in **(A)** Rhode Island (RI) and **(B)** Massachusetts (MA) corals. Point size represents the number of ASVs associated with function that were enriched in either the ambient (orange) or cold (gray) treatments.

Using the inferred functional trait analysis, we observed sulfur cycling associated functions including dark sulfite oxidation, dark sulfur oxidation, dark oxidation of sulfur compounds (Figure 7; Supplementary Figure 4) enhanced in the corals in the cold treatment. Additionally, in the cold treatment corals, there were more ASVs associated with nitrate reduction and fermentation (Figure 7, see Supplementary Figure 4 for which ASVs are associated with which functions over time). In general, we observed more functional traits associated animal parasites or symbionts in ambient treatment corals (Figure 7). ASVs associated with chemoheterotrophy and phototrophy were observed in both treatments.

### 3.7 Aquaria seawater-associated microbial communities

Aquaria seawater microbial alpha diversity differed slightly based on time and treatment, although this was mainly driven by differences in treatments at the start of the experiment between the ambient and cold treatments (Supplementary Figure S5). Seawater microbial communities (Figure S5, Table S4, D0, *p* = 0.006, D1, *p* = 0.09), but did not differ across diversity metrics from ambient treatment seawater microbes (D0, D1, D2, *p* > 0.91). Generally alpha diversity in the water column did not differ between treatments within or across time points (*p* > 0.5, Table S4).

Seawater microbial community dispersion did not vary significantly due to time (Day, *F*_5,32_ = 1.94, *p* = 0.11), treatment (Cold versus ambient, *F*_1,32_ = 0.53, *p* = 0.5) nor their interaction (Day × Treatment, *F*_5,32_ = 1.54, *p* = 0.20). However, seawater microbial community composition differed based on the interaction of time and treatment (*p* < 0.001. Figures S6, S7, Table S5). Pairwise differences suggest fewer compositional shifts between time points in the ambient treatment then the cold treatment as temperatures increased (Supplementary Table S5).

There was some overlap in ASVs detected on the *A. poculata* colonies and in Figure S8. Other bacteria were found occasionally in the seawater, but consistently on corals (*Nitrospina*, *Algicola*, BD1-7 clade), and some were rarely found in the water, but consistently on corals (*Pseudahrensia*, *Ruegeria*).

## 4 Discussion

Here we experimentally manipulated the dormancy of the temperate coral, *Astrangia poculata,* in a controlled lab system. We confirm that quiescence-associated changes in host behavior and in patterns of microbial taxonomic composition and diversity previously observed in field-collected corals (Brown et al., 2022) can be induced and maintained in laboratory conditions by holding corals at low temperatures. Additionally, we found population-level differences in microbial communities, although the two coral populations demonstrated similar overall trends associated with temperature treatments, in terms of diversity, dispersion, and directional community shifts. Furthermore, we demonstrated a persistent shift in the *A. poculata* microbiome composition during dormancy, and as the colonies emerged and recovered from cold-stress-induced dormancy. The observed decline in beta dispersion (Figure 4) is consistent with previously described patterns of microbiome reassembly and holobiont recovery from elevated levels that are a signature of environmental disturbance (Vompe et al., 2024) and this pattern is conserved across individuals.

### 4.1 Host effects: confirming temperature triggers dormancy entry and exit in temperate corals

Our experiment demonstrated that *A. poculata* exposed to 5 °C in the laboratory for 1 month will enter and remain dormant throughout that period. This effect has been previously hypothesized based on field observations (Grace, 2017; Brown et al., 2022) and short-term experiments (Wuitchik et al., 2021). We also demonstrated that entry and exit from dormancy was triggered by temperature, confirming it as an external driver of dormancy for *A. poculata*. We observed little difference between the two populations (Rhode Island and Massachusetts) in the temperatures at which they entered or exited dormancy, suggesting that the temperature trigger for entering/exiting dormancy is conserved across these populations, as has been demonstrated with other thermal traits, such as their thermal optimum (Holliman et al., 2025). Understanding these triggers, and how they may interact with other environmental parameters to influence the incidence and duration of quiescence in *A. poculata*, is a key consideration as winter sea surface warming is occurring in these habitats, as it likely impacts whether corals engage in quiescence and the duration of their quiescence period.

### 4.2 Microbial diversity and beta dispersion shifts associated with emergence from dormancy: short- and long-term recovery

Cold-induced dormancy in *A. poculata* in the laboratory led to a significant decline in microbiome diversity across all tested alpha diversity measures. We observed more ASVs/putative functions associated with putative pathogens, such as *Arcobacter,* in ambient conditions than in the cold/dormancy conditions, consistent with our previous observations in field-collected *A. poculata*, in which a shedding of copiotrophic and pathogen-associated microbes was associated with entry to dormancy (Brown et al., 2022).

Additionally, microbiomes of dormant corals and those re-emerging from dormancy exhibited lower levels of dispersion, relative to those of corals in the ambient treatment. This increase in predictability of composition indicates shared factors that may regulate microbiome composition, resulting in microbiome compositions becoming more similar to one another during dormancy and during re-assembly. These patterns are consistent with previous seasonal surveys in Rhode Island, in which microbial communities sampled from whole coral colonies during the spring exhibited similarly lowered dispersion relative to other months, although it is unknown when the corals emerged from dormancy in that specific year (2017, Sharp et al., 2017). Interestingly, in the current study, as time from cold-induced dormancy increased (i.e., day 57, 2 weeks following exit from dormancy), the initially low dispersion and alpha diversity values increased in the cold treatment and approached values of the dispersion levels of the ambient treatment, suggesting that over the course of microbiome reassembly, there are likely different microbial taxa that colonize individual coral surfaces from the environment, resulting in a less predictable microbiome composition over time.

### 4.3 Re-shuffling of the coral microbiome is persistent after emergence from dormancy

Our results also suggest that microbial communities shift according to treatment and timepoint, even over only 2 days, and this shift in composition persists over time. We found that microbial communities associated with cold-induced dormancy follow similar trajectories: they vary over the time period associated with emergence/recovery from dormancy and do not return to the original community composition, nor do they converge on the community composition in the ambient treatment. Interestingly, we also observed a shift in the ambient corals, although the community was composed of members significantly different from the cold treatment. This is likely due to disturbance associated with the experiment initiation. This result suggests that disturbances in general can lead to persistent microbial remodeling for both the Massachusetts and Rhode Island populations, indicating the *Astrangia* microbiome’s flexibility to change in response to environmental disturbance (Maher et al., 2020; Voolstra and Ziegler, 2020).

Interestingly, corals from MA and RI became more similar to one another following recovery from dormancy, which may be due to colonization of available microbes from the seawater in the experimental conditions, and/or a post-dormancy microbial community’s new state. How this new, post-dormancy state influences coral persistence is unknown, but may be part of the coral’s stress-recovery seasonal cycling (Glasl et al., 2016; Sharp et al., 2017), and indicative of acclimatization (Reshef et al., 2006; Vompe et al., 2024), and/or a reflection of priority effects following a disturbance (Debray et al., 2022). Continued work on the microbial community succession after dormancy will help to inform what microbial taxa and functions are important for microbiome re-assembly following environmental disturbance. However, it is unknown if this indicates that these populations are well positioned to withstand new seasonal climate regimes (i.e., warmer winters) or if winter warming will impact long-term flexibility.

### 4.4 Nutrient cycling and protective roles of the microbiome during dormancy and following emergence

The mucus microbiome of dormant *A. poculata* appears to be involved in sulfur cycling, as suggested by increases in *Sulfitobacter* throughout the dormant period and early recovery (here and in Brown et al., 2022). Sulfitobacter is a major sulfite-oxidizing bacterial group from the Roseobacter clade found in temperate corals (Brown et al., 2022), deep sea corals (Lin et al., 2023) and tropical corals (Raina et al., 2009). Some *Sulfitobacter* can convert DMSP to DMS (Zeng et al., 2016) and these compounds are hypothesized to play major roles in structuring coral microbial communities (Raina et al., 2010). Other members of the Sulfitobacter group can convert DMSP to tropodiethetic acid (TDA), which has anti-*Vibrio* and protective roles for algal cells (Seyedsayamdost et al., 2011) and is thought to play a role in host protection in many animal-algal and algal-bacterial associations. Additionally, *Sulfitobacter* can support microalgal growth and provide pathogen protection, possibly aiding the resident endosymbiotic algae associated with the corals, or contributing to anoxygenic photosynthesis (Yang et al., 2021; Beiralas et al., 2023; Li et al., 2022). Thus, it is possible that *Sulfitobacter* may play an important role as an early colonizer and regulator of the microbiome as it re-assembles after quiescence.

Nitrogen-cycling microbes are also associated with *A. poculata* dormancy. These included Colwellia and Rhizobacteriacae, which are associated with nitrogen cycling in tropical corals (Figures 6, 7; Supplementary Figure S4). We previously observed the same pattern in wild *A. poculata* in during and following emergence from dormancy: an increase in bacterial groups associated with nutrient cycling (Brown et al., 2022). We expect these shifts to be associated with nutritional provisioning for the host during recovery. Because we conducted repeated sampling of the mucus on the corals and tracked their performance throughout the experiment, we were unable to analyze microbial members that are likely located within coral tissues and not in the mucus microbiome, such as *Nitrosopumilus*, an archaeal ammonia oxidizer previously consistently documented in *A. poculata* (Sharp et al., 2017; Brown et al., 2022) and other tropical corals (Siboni et al., 2008). Thus, although we did not capture consistent *Nitrosopumilus* using mucus sampling, it is possible that it remains in the coral tissues.

Combined, the taxonomic shifts and their putative functions suggest that the shifts in the coral microbiome during recovery from cold-induced dormancy may play a role in augmenting nutritional provisioning and play a protective role for the holobiont, directing the re-assembly of a stable, predictable microbiome after emergence from quiescence. However, within this experimental design, it is not clear if the mucus-associated microbiome shifts are a response to the temperature shifts, to the holobiont quiescence, or a combination of both factors.

### 4.5 Host control of the microbiome, even during dormancy

Throughout the course of this experiment, the *A. poculata* mucus microbiome was consistently different from the seawater microbiome. Although the seawater might act as a reservoir and source for some colonizing bacteria (e.g., *Sulfitobacter*), the seawater microbiome alone cannot explain the variation observed on coral surfaces (Supplementary Figure S8). Coral and seawater microbial communities differed in presence or relative abundance of taxa, suggesting the coral holobiont-driven regulation of the microbiome, even during quiescence, potentially by the microbes themselves. However, the differences in seawater microbial communities between our aquarium systems and the wild environments may influence some of the observed differences in microbiome composition of lab and diversity in field and laboratory dormancy studies.

### 4.6 Population differences in coral microbial communities

Although populations from MA and RI demonstrate similar patterns in response to dormancy, including similarity in genera-level bacterial changes, initial microbial community composition associated with Massachusetts and Rhode Island corals differed. Additionally, coral microbial diversity and dispersion varied between populations. Corals were maintained in common aquaria conditions for at least 2 months before the start of the experimental measurements, highlighting the prior environment or host-mediated selection and maintenance of microbial communities of these two populations. Placing corals in experimental units led to initial shifts in community composition across all corals, including the decline in the variation in microbial communities between populations. Following treatment application, differences in the microbiome across populations emerged following dormancy. Previous work suggests population-level differences in host physiological performance (Holliman et al., 2025), which may then influence or be influenced the microbiome. The variation we observed in microbial response to treatments could reflect subtle differences in host selection or ability to shift their microbiome, which can vary across and within species of corals (e.g., microbial flexibility, Voolstra and Ziegler, 2020).

### 4.7 Coral emergence from dormancy is a useful proxy for tropical coral recovery

*Astrangia poculata* is a useful experimental system for studying the physiology and ecology of coral-microbe symbiosis (Puntin et al., 2022), with particular value as a host species that is closely related to many tropical scleractinian corals with a taxonomically similar microbiome to many tropical corals. The findings in this study, taken together with recent *A. poculata* microbiome studies, suggest that cold-induced quiescence in *A. poculata* is an excellent model for studying microbiome dynamics following general environmental disturbances, including thermal and nutrient stress, bleaching, and disease, in tropical corals. A common signature of stress in animal microbiomes is increased beta dispersion during a disturbance event (Zaneveld et al., 2017) and occasionally an increase in alpha diversity (Brown et al., 2021), and thus following the event, during recovery, there is a decrease in dispersion, associated with that recovery. For example, tropical coral microbiomes exhibit declined diversity following heat stress (Maher et al., 2020), and corals that experience and tolerate stress show decreases in dispersion (Vompe et al., 2024) during recovery as we have observed in *A. poculata* in this study and in previous studies in *A. poculata* microbiomes surrounding winter dormancy (Sharp et al., 2017; Brown et al., 2022). The patterns observed in this study are consistent with constriction of the microbiome associated with general stress-recovery responses of corals. Additionally, coral acclimatization to stressors via microbial community shifts in composition is a core tenet of the coral probiotic hypothesis (Reshef et al., 2006), with some support that following heat exposure or probiotic addition, coral microbial communities shift in response to temperature stress (Ziegler et al., 2017, 2019; Santoro et al., 2021). Our work also identifies *Sulfitobacter*, known to regulate tropical coral community structure (Raina et al., 2009, 2010), as a group that may play an important early role in coral microbiome recovery. Identifying the bacterial taxa involved in the predictable reassembly and remodeling of the microbiome after an environmental disturbance, as shown here in experimentally induced quiescence and emergence from quiescence, can inform the development of new interventions and strategies for tropical coral recovery and resilience to global environmental change.

## 5 Conclusion

Dormancy is a response to stress that many species use to adapt to extreme fluctuations in environmental conditions, and emergence from dormancy is analogous to recovery from disturbance. Thus, understanding dormancy-associated microbiome changes can provide key insight into how microbes mediate holobiont response, resilience, and recovery to environmental disturbances. Our work supports that *A. poculata* entry and exit into quiescence can be controlled by temperature, and microbial patterns of diversity and composition on a bulk scale are consistent between lab-induced and field quiescence: a decline in diversity, followed by a predictable re-structuring of the microbiome during emergence from quiescence. We suggest that the microbiome, in addition to the host, may influence this re-structuring, through sulfur and nitrogen cycle-associated microbes. We highlight that for *Astrangia*, annual seasonal quiescence and emergence from quiescence leads to predictable microbial responses that are conserved across nearby populations. In this study we have identified bacteria, including *Sulfitobacter*, that may be significant primary colonizers of the mucus layer involved in mediating reassembly of the microbiome following disturbance. Our work highlights that dormancy in *A. poculata* is a useful model to study microbial dynamics associated with stress and recovery and to identify key microbial taxa involved in the recovery of animal microbiomes from environmental disturbance.

## Data Availability

The datasets generated for this study can be found in the NCBI SRA Database (https://www.ncbi.nlm.nih.gov) under BioProject PRJNA1440488; accession numbers SRX32740007-SRX32740256.
