# Supplementary material for: Controlled temperature-induced dormancy leads to predictable microbial recovery in the temperate coral microbiome
Source: Front Microbiol. 2026 May 7;17:1799468. doi: 10.3389/fmicb.2026.1799468 (PMC13190580; doi:10.3389/fmicb.2026.1799468)
Supplement: Supplementary file 1 [file Supplementary_file_1.docx]

Supplementary Material

Controlled temperature-induced dormancy leads to predictable microbial recovery in the temperate coral microbiome

Anya L. Brown ^1,2*^, Meriel J. McGovern ^3,4,^ Alicia Schickle ^3^, Koty Sharp^3^ and Amy Apprill^2^

^1^Bodega Marine Lab, University of California, Davis, Bodega Bay, CA, United States,

^2^Woods Hole Oceanographic Institution, Woods Hole, MA, United States,

^3^Roger Williams University, Bristol, RI, United States,

^4^Princeton University, Princeton, NJ, United States

Supplementary Figures

**Supplementary Figure 1**. A) Coral-associated mucus and B) aquaria seawater microbial sequence rarefaction curves.


**Supplementary Figure 2**. Probability of time to dormancy. Panels represent corals in the ambient (orange) and cold (gray) treatments from Massachusetts (A,B) and Rhode Island (C,D). Left panels (A,C) are time until corals stopped responding to touch, and right panels (B,D) represent time to polyp retraction. Results demonstrate rapid entrance into dormancy for cold treatment corals. P-values on each figure represent significant differences in survival probability between ambient and cold treatments.

**Supplementary Figure 3**. Probability of time to emergence from dormancy. Panels A and B represent corals from Massachusetts and panels C and D represent corals from Rhode Island. Panels A and C represent corals responding to touch following the rise of temperatures (starting on Day 37). Panels B and D represent corals unfurling their tentacles (less than 100% retraction). P-values on each figure represent significant differences in survival probability between ambient and cold treatments.

**Supplementary Figure 4**. Putative functions associated with ASVs that were significantly different according to DeSeq results. Enrichment differences are based on log2fold changes of ASVs by time and population between treatments. Note, some genera are found multiple times above because they are associated with multiple putative functions.

Seawater microbiomes

**Supplementary Figure 5**. Alpha diversity as Hill numbers a) D^o^ (Richness), b) D^1^ (exponiated Shannon) and c) D^2^ (Inverse Simpson) associated with seawater samples collected at each time point in both the ambient (orange) and cold (gray) treatments.

**Supplementary Figure 6**. Mean ± SE of beta dispersion of seawater microbial communities. Small points are raw data. Colors indicate differences in treatments (Ambient and Cold treatments). Dispersion did not vary significantly due to time (F_5,32_ = 1.94, p = 0.11), treatment (F_1,32_ = 0.53, p = 0.5) nor their interaction (F_5,32_ = 1.54, p = 0.20).

**Supplementary Figure 7**. NMDS comparison of seawater microbial communities in the ambient (A) and cold (C) treatments. Communities differ based on the interaction between treatment and time.

**Supplementary Figure 8**. Significantly differentially abundant microbial taxa in corals in the Ambient and Cold treatments in both seawater and coral sample types. Color indicates treatment. Size of point represents relative abundance. Absence of a point indicate that ASVs in that genera are absent in that treatment or sample type.

**
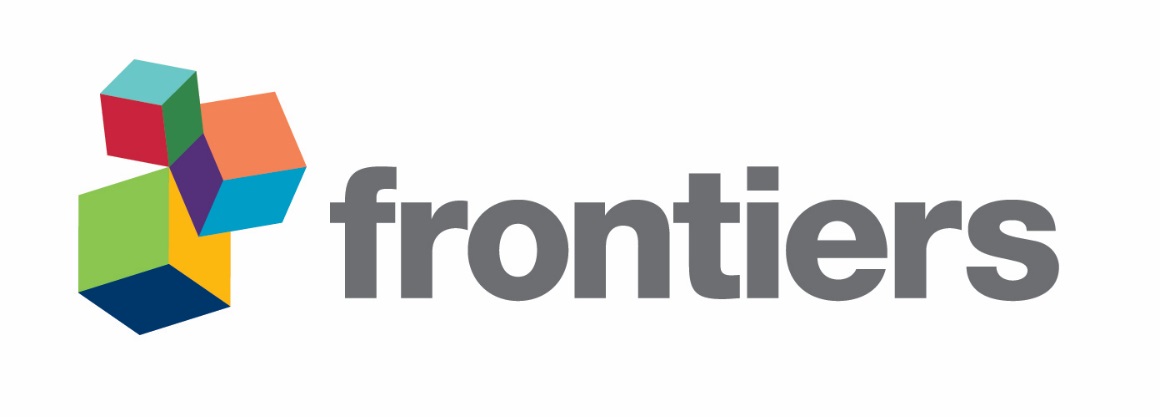
**
